# Form and Function of Early Neolithic Bifacial Stone Tools Reflects Changes in Land Use Practices during the Neolithization Process in the Levant

**DOI:** 10.1371/journal.pone.0042442

**Published:** 2012-08-08

**Authors:** Richard W. Yerkes, Hamudi Khalaily, Ran Barkai

**Affiliations:** 1 Department of Anthropology, Ohio State University, Columbus, Ohio, United States of America; 2 Israel Antiquities Authority, Jerusalem, Israel; 3 Department of Archaeology, Tel-Aviv University, Tel-Aviv, Israel; New York State Museum, United States of America

## Abstract

For many, climate change is no longer recognized as the primary cause of cultural changes in the Near East. Instead, human landscape degradation, population growth, socioeconomic adjustments, and conflict have been proposed as the mechanisms that shaped the Neolithic Revolution. However, as Bar-Yosef noted, even if there is chronological correlation between climate changes and cultural developments, what is important is to understand how Neolithic societies dealt with these improving or deteriorating environments. Changes in bifacial stone tools provide a framework for examining some of these interactions by focusing on changing land use practices during the Neolithization process. The results of microwear analysis of 40 bifacial artifacts from early Pre-Pottery Neolithic (EPPNB) levels at Motza in the Judean hills document changes during the PPNA–PPNB transition at the onset of the Levantine Moist Period (ca. 8000 cal B.C.) when conditions for agriculture improved. EPPNB villagers added heavy-duty axes to a toolkit they had used for carpentry and began to clear forests for fields and grazing lands. Sustainable forest management continued for the duration of the PPN until the cumulative effects of tree-felling and overgrazing seem to have led to landscape degradation at end of the Pre-Pottery Neolithic C (PPNC), when a cold, dry climatic anomaly (6600–6000 cal B.C.) may have accelerated the reduction of woodlands. Early PPNB components at sites like Motza, with data from nearly five millennia of Neolithic occupations, show how complex hunter–gatherers and early food producers were able to establish sustainable resource management systems even as climate changed, population increased, and social relations were redefined.

## Introduction

The transition from mobile Paleolithic to sedentary Neolithic life ways may be the most significant behavioral “revolution” in human prehistory. When hunter-gatherers domesticated plants and animals and congregated in larger settlements, they were laying the foundations for the development of complex urban societies and our modern world system. The Levant has long served as a laboratory for understanding these significant changes in human activities and perceptions. However, the shift to Neolithic lifeways is now viewed as a slow process rather than a rapid revolution, and thus the term *Neolithization* is used to describe the set of events that led to the emergence of social complexity in the Levant [1], [2], [3].

Once considered the forcing mechanism for this process, climate change is no longer recognized as the primary cause. Instead, human landscape degradation, population growth, socioeconomic adjustments, and conflict have been proposed as the forces that changed the lives of terminal Pleistocene foragers. In the Levant, the transition to agriculture was revolutionary, but it was a slow, complicated process that played out on different landscapes under changing climatic conditions between ca. 9700 and 6200 cal B.C. [1], [2], [4], [5], [6]. Recent refinements in proxy data for Early and Middle Holocene climates revealed that rapid climate change (RCC) cooling events are correlated with some cultural transformations [4]. However, as Ofer Bar-Yosef [7] noted, even if there is chronological correlation between climate changes and cultural developments, what is important is to understand how Neolithic societies responded to improving or deteriorating environments. One way of examining some of the ways that complex hunter-gatherers and early food producers responded and “constructed new niches” [8] is to study the changes in the form and function of their bifacial lithic tools. The production and use of new tool types during the two phases of the Pre Pottery Neolithic (PPNA-PPNB ca. 9700-6600 cal B.C.) and the Pre-Pottery Neolithic-Pottery Neolithic (ca. 6600-6000 cal B.C.) transition may have been a response to a combination of environmental, economic, and social changes. Studies of these changes in technology and tool use can lead to broader considerations of shifts in ideology, settlement patterns, and social organization, and help evaluate competing models that emphasize different causal factors, ranging from human landscape degradation, population growth, socioeconomic adjustments, to the organization of ritual and warfare. In this study, we focus on the earlier transition (PPNA-PPNB) and show how new kinds of bifacial tools were manufactured and used when the forests were opened for agriculture and more wood was needed for larger structures and for fuel. We propose that our modern perceptions of wood as a renewable, managed resource had its origins in the Neolithic period in the Levant. Pleistocene foragers did not need to make axes to clear the forests or fell trees for construction and fuel. These tools were needed only when the Neolithization process began.

### The Long Winding Road to Farming in the Levant

It has been suggested that at the end of the Paleolithic, during the warm and wet climate of the Bølling-Ållerød instertadial (ca. 12,550-11,050 cal B.C.), sedentary Early Natufian hunter-gatherers started down the road to agriculture only to be interrupted by the return of cold and dry conditions during the Younger Dryas, ca. 10,950-9550 cal B.C. [9], [10]. While many believe that the increased mobility of the Late Natufian foragers was a response to deteriorating climate, alternatives to this climate-forcing model for changes in subsistence and mobility strategies at the end of the Pleistocene in the Levant have been proposed that employ niche construction and resilience theory [8], [11] – but a detailed discussion of these theories is beyond the scope of this study. Nonetheless warmer and wetter conditions after the Younger Dryas with forest expansion and higher biodiversity seem to have favored the re-establishment of more sedentary settlements during the PPNA period (9700 to 8550 cal B.C.; refs. 1, 2, 5, 6, 10). However, the PPNA complex hunter-gatherers established larger, more permanent settlements with new types of houses and tools and began the gradual process of Neolithization that created real agricultural systems during the PPNB (8550-6750 cal B.C.). While both the Early Natufian and PPNA societies may have been “primed” for agriculture [12], environmental, demographic, social, and ideological conditions at ca. 8300 cal B.C. seem to have been more favorable for the development of food production than they were 4000 or 1400 years earlier [13], [14]. During the Levantine Moist Period (LMP, 8000-6600 cal B.C.), Middle and Late PPNB tribes living in large villages added more domesticates to their broad-spectrum foraging regime and devoted more time to clearing fields, storing their harvest, and corralling animals [1], [4], [6], [7].

The timing of the onset of the actual Neolithization process led some to suggest that favorable LMP climate was a major factor [1], [4], but in another model, rapid population growth (e.g., the Neolithic demographic transition, NDT, or agricultural demographic transition, ADT) were the underlying causes. These demographic models attribute the growth of PPNA villages – which were ten times larger than Natufian base camps, but with smaller catchments – to reduced birth spacing and increased fertility, due to decreased mobility and the use of cereals to wean children. In the models, the initial stage of rapid population increase is followed by a second stage when mortality increased from the spread of diseases in the autonomous villages [15], [16]. Most PPNA villages were abandoned after 200–400 years, but there is no evidence of epidemics. Cycles of village nucleation and dispersal may have been due to internal conflicts between tribal segments, or a turning away from ambitious leaders [13], [14], [17], but evidence for these processes is also elusive.

It is more common to attribute the abandonment of large PPNA (and later PPNB) villages to increasing internal social tensions, rapid deleterious climate change, or landscape degradation (erosion, salinization) from felling too many trees, overgrazing, and over use of agricultural fields [1], [7], [18], [19], [20]. While there are some correlations between cold and dry climate anomalies and calibrated radiocarbon dates for the abandonment of some PPNA/EPPNB settlements at 8200-8000 cal B.C., and larger LPPNB villages at 6600-6000 cal B.C. [1], [4], [7], others see human landscape degradation as the main cause [18], [19], [20]. In the absence of detailed landscape reconstructions, another line of evidence, changes in the form and function of bifacial lithic tools, may provide insights into land use practices during the PPNA–PPNB and Pre-Pottery Neolithic-Pottery Neolithic transitions.

### Changes in Bifacial Tool Production in the Levant

Very few bifacial tools were made between the Lower Paleolithic (ca. 1.4-0.2 million years ago) and Epipaleolithic (23,000-14,000 cal B.C.) periods in the Levant. After this hiatus of some 185,000 years, enigmatic bifacial Natufian “picks” were produced at the end of the Pleistocene, but the first wood-working tools were not made until the PPNA period (9700-8550 cal B.C.). These include flint bifaces with symmetrical (biconvex) longitudinal cross-sections and straight or convex distal edges, classified as *axes*, rather than *adzes.* Adzes have asymmetrical longitudinal cross-sections and rectangular or triangular outlines. The first adzes are found in Levantine PN lithic assemblages, replacing axes as heavy-duty wood-working tools [21], [22]. Chisels are smaller, narrower, bifacial tools usually with asymmetrical longitudinal cross-sections and oval outlines, or contracting proximal and/or distal ends.

The edges on flaked PPNA flint axes and chisels were shaped with transverse blows, but in addition to these flint *tranchet* axes, ground and polished axes made of limestone, distinctive “greenstones,” and other coarse-grained lithic materials were also manufactured. During the PPNB period (8550-6600 cal B.C.), the edges of larger flaked flint axes and chisels, were shaped by grinding and polishing rather than transverse blows, and groundstone axes are less prominent. During the Pottery Neolithic period (PN, 6600-4500 cal B.C.), more versatile flint adzes, hafted perpendicular to their handles (like hoes), with ground and polished edges replaced the flaked flint axes – and were later replaced by metal axes during the Chalcolithic and Bronze Ages [21], [22]. Ongoing technological and microwear studies of samples of Neolithic bifacial tools from the southern Levant revealed that while nearly all were used for wood-working, it was possible to distinguish between bifacial tools used for heavy duty tree-felling and log splitting from smaller bifaces used for carpentry and lighter duty wood-working [22], [23].

**Figure 1:**
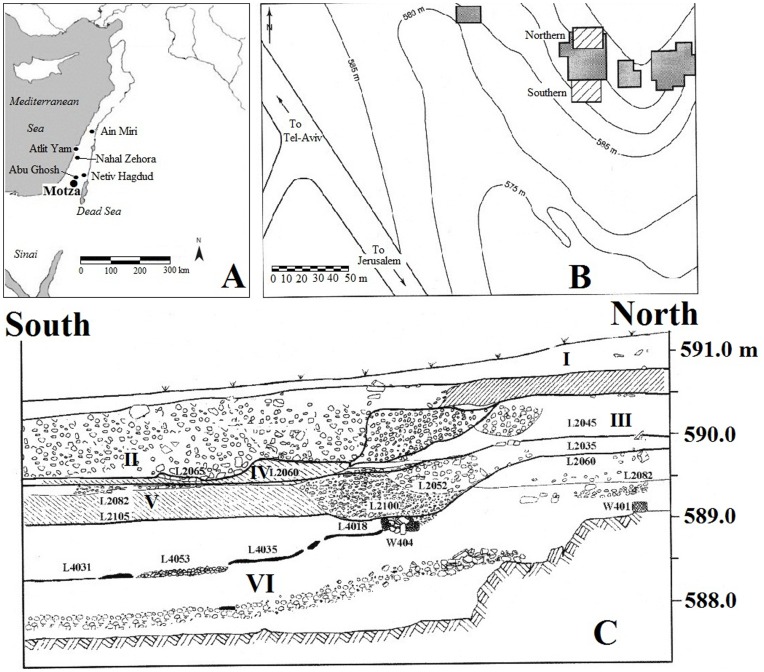
A: Neolithic sites mentioned in the text, B: Locations of Northern and Southern blocks at the Motza site excavated by HK on behalf of the Israel Antiquities Authority in 2002 and 2003, C: Main stratigraphic section for the 2002–2003 excavations at Motza. VI is the ca. 2 m thick EPPNB layer exposed directly above bedrock [24].

While samples from PPNA (NetivHagdud), late PPNB (‘AinMiri), PPNC (Atlit-Yam), and PN (NahalZehora I and II) sites in the southern Levant have been examined [22], [23], the sample of 40 bifacial chipped and groundstone artifacts from the early PPNB Layer VI at the Motza site presented here is of particular interest, since it marks the PPNA–PPNB transition in the southern Levant (Fig. 1). This transition is of special significance since it marks the emergence of the first agricultural communities in the Levant. The PPNA economy was still based on hunting and gathering, but larger settlements were established. Another significant practical and conceptual change that took place during the PPNA–PPNB transition is the shift in house construction from the circular or oval form that was common during the Natufian and PPNA periods to the typical rectilinear form of the PPNB and later periods in the Levant. These Neolithic developments in economy and architecture are fully evident during the middle phases of the PPNB (MPPNB), but make their first appearance during the EPPNB. Since MPPNB societies living in rectangular structures made use of heavy polished flint axes for tree-felling and heavy-duty wood working activities, while their PPNA hunter-gatherer predecessors constructed round structures using lighter, smaller carpentry tools (e.g. *tranchet* axes and chisels), changes in the form and function of bifacial tools document how Levantine societies were creating new environmental, economic, and social conditions during the Neolithization process.

### The Motza Site in the Judean Hills

Early, Middle and Late Pre-Pottery Neolithic B, Pottery Neolithic, Bronze Age, and Iron Age components were identified at the ca. 9 ha. Motza site, located 5 km west of Jerusalem, and 5 km SE of the Abu Gosh Neolithic site (Fig. 1). The site was inhabited by Neolithic groups for nearly five millennia. Sixteen calibrated radiocarbon dates from EPPNB Layer VI (Figure 1C) ranged from 8600-8200 cal. BC, similar to dates from other EPPNB sites in the southern Levant [24], [25]. The ca. 2 m thick Early PPNB occupation Layer VI contained substantial round and rectangular stone and mud brick structures (some with lime plastered floors), and at least two thick stone walls. There is a rich faunal assemblage and a sizable collection of bone tools from secure, well-dated EPPNB contexts in Layer VI. Several human burials were exposed in that layer, and some small animal and human figurines were recovered [24].

Over 91,000 lithic artifacts were recovered from EPPNB Layer VI at the Motza site, and a 10% sample of 9,384 were described in the preliminary report [24], including 202 projectile points, 463 other formal blade or flake tools, 67 cores, two hammerstones, and 30 bifacial tools. The microwear sample included 20 of these bifacial tools (67%). All of the groundstone axes (n = 4), flaked stone *tranchet* axes (n = 6) and *tranchet* chisels (n = 7) were examined for technological and microwear traces. The *tranchet* axe with a polished bit, an unpolished flaked stone chisel, and a recycled biface (probably an axe fragment) from the Layer VI excavations were also examined (Table 1), along with 20 *tranchet* spalls that were removed when *tranchet* axes and chisels were manufactured and resharpened (35% of the recovered spalls).

**Table 1:** Motza EPPNB, Summary of Microwear and Technological Analysis.

*Groundstone axes*	Edge	Weight	Edge			Worked	Other		
SN	Locus	G.L.	Shape	grams	fractures	Used	Motion	Material	Notes
1	4052	101.3	convex	260					*no visible wear traces*
2	2136	75.2	convex	109		**X**	chopping	wood	*very light use*
3	4070	106.0	convex	183					*not used*
4	5059	81*	straight	233	some nicks	**X**	chopping	wood	*fragment, very light use*
***Tranchet axes***									
7	5052	58.3	convex	29	battered	**X**	chop/plane	wood	*haft traces*
11	5040	73.8	convex	30	retouch?	**X**	planing	wood	*hafted, resharpened?*
13	4062	60.0	convex	24	some nicks	**X**	planing	wood	*“humped,” light use*
**15**	**5016**	**59.0**	**convex**	**25**	**battered**	**X**	**chopping**	**wood**	***bit is polished, hafted***
16	5008	79.0	straight	39	7 feather	**X**	chop/plane	wood	*hafted, nicked corners*
32	4050	74.5	straight	32	8 feather	**X**	chop/plane	wood	*haft traces*
34	5040	61.9	convex	29	battered	**X**	chop/plane	wood	*cortex, haft traces*
***Tranchet chisels***									
5	5060	51.3	straight	17	corner damage	**X**	planing	wood	*light use*
6	4050	51.7	convex	13	some nicks	**X**	planing	wood	*haft traces, light use*
8	5056	52.5*	straight	19	some nicks	**X**	planing?	wood	*fragment, light use*
10	4062	36.0	straight	9	8 feather				*no visible wear traces*
12	5062	53.1	convex	18	some nicks				*fragment, not finished?*
14	4014	51.3*	straight	10	10 feather	**X**	planing	wood	*fragment, intense use*
17	5083	52.4	straight	13	some nicks	**X**	planing	wood	*hafted, double-edged*
***Other chisel and axe***									
9	4052	59.4	straight	14	3 step	**X**	planing?	wood?	*hafted, cortex, light use*
33	5067	59*	n/a	72	n/a	?	polishing	stone	*recycled axe fragment*
***Tranchet spalls***									
18	4070	28.0	straight	9	retouch				*technological traces*
19	4070	18.0	straight	2	retouch	**X**	chop/plane	wood	*very light use*
20	5027	36.5	convex	22	retouch				*cortex, tech. traces*
21	4070	11.0	straight	1	retouch				*lustrous, abrasion*
22	4070	8.5	straight	1	retouch				*technological traces*
23	4070	34.0	convex	26	retouch				*no visible wear traces*
24	4070	19.0	convex	3	retouch	**X**	chop/plane	wood	*some abrasion*
25	4070	17.8	convex	4	some nicks	**X**	chop/plane	wood	*lustrous, light use*
26		14.5	straight?	7	battered	**X**	chop/plane	wood	*lustrous, moderate use*
27	5040	36.3	convex	9	retouch				*no visible wear traces*
28	5040	13.0	convex	2	retouch	**X**	chop/plane	wood	*lustrous, abrasion*
29	5053	13.8	straight	1	retouch				*technological traces*
30	5053	11.0	straight?	1	retouch	**X**	chop/plane	wood	*lustrous, abrasion*
31	5053	15.0	straight	3	retouch				*technological traces*
35		27.8	straight	1	retouch				*technological traces*
36		37.0	convex	5	retouch				*cortex, tech. traces*
37		47.0	convex	4	retouch				*technological traces*
38	4070	44.8	straight	12	retouch				*coarse grained flint*
39	4070	24.3	convex	1	retouch				*no visible wear traces*
40		34.8	convex	2	retouch				*technological traces*

**G.L.**: greatest length in mm; **Edge Fractures**: type of flake scars along cutting edge; battered: many edge fractures and flake scars along bit; **Used**: there is microwear or macrowear evidence for utilization; **Motion**: how the tool was used, heavy chopping, or lighter wood-working (e.g., carpentry); **Worked Material**: the type of material modified by the tool; *cortex*: some of the original exterior surface of the rock can still be seen on the artifact; *fragment*: tool is not complete; *haft traces, hafted*: wear traces suggest that the tool was attached to a handle; “*humped*:” failure to thin biface properly has left a thick hump on one or both faces; *resharpened*: the tool seems to have been retouched to restore a sharp edge. The tranchet axe with the polished bit (SN 15) is shown in **boldface**.

**Notes on contexts**: one of the groundstone axes (SN 3) was found in the same locus (4070) where nine *tranchet* spalls were recovered. A calibrated radiocarbon date on bone from this Locus (4070) was 8336–8284 cal BC (1σ). A flint *tranchet* axe (SN 32) and a flint *tranchet* chisel (SN 6) were found in Locus 4050 where parts of a secondary burial feature that included at least five individuals were exposed, and a broken greenstone figurine that was recycled as a pendant was also recovered. A flint *tranchet* axe (SN 13) in Locus 4062 was associated with a calibrated radiocarbon date on bone (8304–8278 cal BC, 1σ). Two flint *tranchet* axes (SN 11 and 34) and two *tranchet* spalls (SN 27 and 28) were all found in Locus (5040). Three *tranchet* spalls (SN 29, 30, and 31) came from Locus 5053, and one came from Locus 5027 (SN 20).

## Results

Axes in Motza EPPNB Layer VI were small and light, and chisels were even smaller – like earlier PPNA types (mean weight of PNNA tranchet axes was <40 grams [21], [24]). Polished flint axes and chisels from Levantine PPNB sites are larger and heavier. Mean weight of PPNB axes is 120 g), and their edges were shaped by polishing rather than by *tranchet* blows [21], [22].

The groundstone axes in the EPPNB layer at Motza also resemble the PPNA types, and their presence, along with the *tranchet* axes and chisels, Khiam points, and bladelets, shows continuity in stone tool production techniques [21], [22], [23], [24].

**Figure 2:**
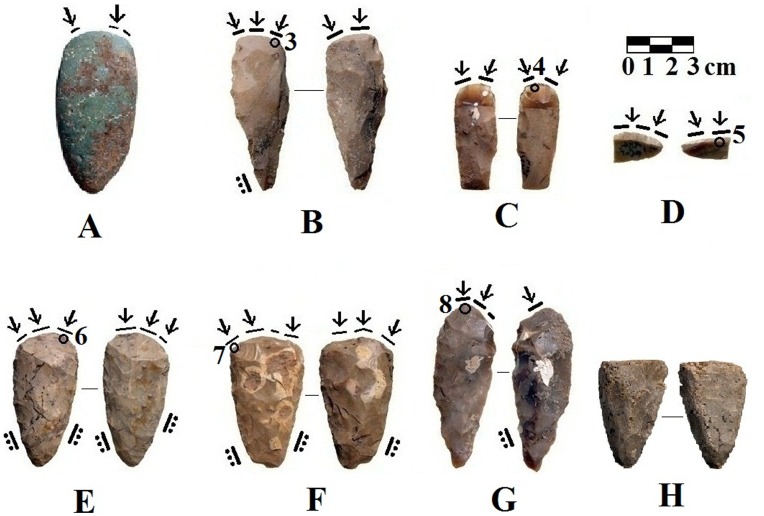
Some utilized Bifaces and a *tranchet* spall from EPPNB layer VI at Motza. Ventral face to the left, dorsal face to the right, black lines show the extent of the wood-working microwear traces, arrows show the orientation of the polish and striations, lines with dots show location of hafting traces. (*A*) Polished greenstone axe from SN 2, Locus 2136, Basket 40013, made of green stone, with weakly-developed wood working traces and slight edge damage along distal edge. (*B*) flint *tranchet* axe, SN 32, Locus 4050, Basket 41007. Circle shows the location of Fig. 3 Right. (*C*) flint *tranchet* chisel N19c, SN 14, Locus 4014, Basket 40211. Circle shows the location of Fig. 4 Right. (*D*) *tranchet* spall, SN 26, Basket 40309. Circle shows the location of Fig. 5. (*E*) **flint** ***tranchet*** **axe resharpened by polishing**, SN 15, Locus 5016, Basket 50135. Circle shows the location of Fig. 6. (*F*) flint *tranchet* axe, SN 34, Locus 5040, Basket 50437. Circle shows the location of Fig. 7. (G) flint *tranchet* axe K17c from Motza, SN 11, Locus 5040, Basket 50479. Circle shows the location of Fig. 8. (*H*) base of a recycled flint axe, SN 33, Locus 5067, Basket 50730 with worn lateral edges and worn dorsal and ventral faces. Some of these surfaces have large patches of stone-on-stone polish. It seems to have been re-used as a stone polishing tool.

However, changes in lithic artifact production are also evident in the EPPNB assemblage. While most of the flint axes and chisels are the same size and weight as PPNA types, and also had cutting edges shaped by the *tranchet* technique, the edge of one axe (SN 15, Fig. 2E) that was originally shaped by *tranchet* blows,had been polished – the standard procedure for sharpening PPNB axes [21]. This is the only example we are aware of where two different techniques were used for shaping the working edge of a Neolithic axe. This typical *tranchet* axe was originally produced in the PPNA fashion, and its working edge was sharp, but fragile. *Tranchet* axes are normally resharpened and maintained by the removal of *tranchet* spalls. In fact, one of the advantages of the *tranchet* sharpening technique is its efficiency. The entire edge is rejuvenated with two transverse blows. Indeed, resharpening *tranchet* axes by the removal of *tranchet* spalls was practiced both at PPNA and EPPNB sites in the southern Levant [21], [22], [23]. We suggest that someone decided to resharpen this particular *tranchet* axe from Motza (SN 15) by grinding and polishing its edge, rather than by removing *tranchet* spalls. The polished working edge would be stronger and more durable, but not as sharp. Flaking and damage to the resharpened edge indicate that after it had been polished, this axe was used for tree-felling and/or log splitting. Most *tranchet* axes were not used for these heavy-duty wood-working tasks (Table 1).

This is the earliest and only example of an EPPNB flint axe with a working edge that was shaped by both the *tranchet* and the polishing techniques. It is most probable that the axe was used in both its *tranchet* and polished forms, and that while it may have initially been used for carpentry, damage to the fragile *tranchet* edge from heavier use may have led the axe-user to grind the and polish the flint edge to produce a more durable edge. This was the technique that had been used to smooth the edges of PPNA and EPPNB groundstone axes [21], [22], [23]. This axe shows the replacement of the *tranchet* technique by the polishing technique during the EPPNB, a technological adjustment that led to the production of stronger and more durable working edges intended for tree-felling and heavy-duty wood working tasks. It seems that the first heavy-duty axes were small EPPNB flint *tranchet* types that were resharpened by polishing their edges. In the LPPNB and PPNC, large flint axes produced with polished edges were only used for tree-felling and heavy duty wood-working [21], [22], [23].

This was not the only innovation recorded in the EPPNB levels at Motza. The cache of 58 naviform bidirectional blades found in Layer VI shows that Motza knappers were already using this sophisticated PPNB production technique [24].

Microwear traces on a complete green stone axe (SN 2) and a broken groundstone axe (SN 4) from the EPPNB layer showed that they were used for wood-working (Table 1; Fig. 2A). There were no wear traces on two other groundstone axes, and they might have been produced for symbolic purposes, as has been suggested for PPNA groundstone axes [21], [22], [23]. The bits of the utilized groundstone axes did not have the edge-damage and impact fractures seen on groundstone and flint axe replicas used to fell trees and split and notch logs [26]. Similar heavy wood working traces are also common on MPPNB and LPPNB polished flint axes [21], [22]. The groundstone axes from Motza seem to have been used to plane and shave wood and clear brush, but not for heavier work like felling trees and splitting large logs. In any case, the fact that two of the polished stone axes from Motza were used in wood-working might reflect a deviation from the PPNA pattern where they served more as symbols while *tranchet* flint axes were made and used for wood-working [21], [23]. The fact that one of the utilized polished stone axes is made of exotic greenstone is also noteworthy. Bar-Yosef Mayer and Porat [27] reported that ornaments made of green stones do not appear in the Levant until the Neolithic. They suggest that green was associated with vegetation, rain, fertility, virility, and strength, and that PPNA hunter-gatherers (and PPNB farmers) obtained green stone from sources quite far from their settlements. These results show that the need for heavier tree-felling bifaces began in the EPPNB as forests were opened to improve the habitat of wild plants and animals, and more wood was need for the new rectangular Neolithic structures. More wood was also needed for the complicated limestone burning process used to make plaster for thick house floors and statues, and probably to build pens for domesticated animals.

**Figure 3:**
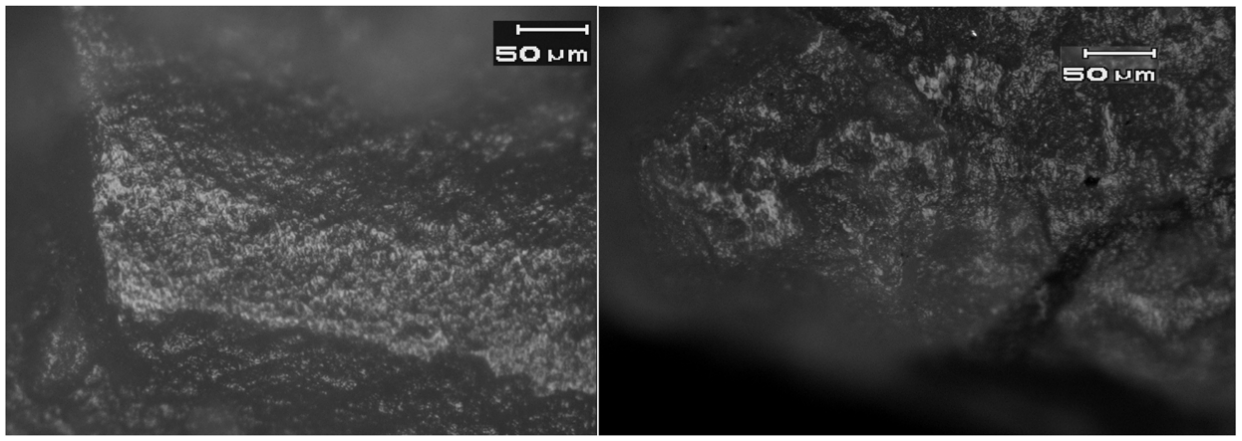
Left: Bright, domed wood working microwear on experimental biface used to plane and smooth wood. **Right**: Bright, domed wood-working microwear traces on the bit of a small EPPNB *tranchet* axe from the Motza site (SN 32, Locus 4050, Basket 41007). See Figure 2B for location. Bit edge is at the bottom of the photomicrograph. Magnification is 187.5x, and scale is 50 microns for both images.

**Figure 4:**
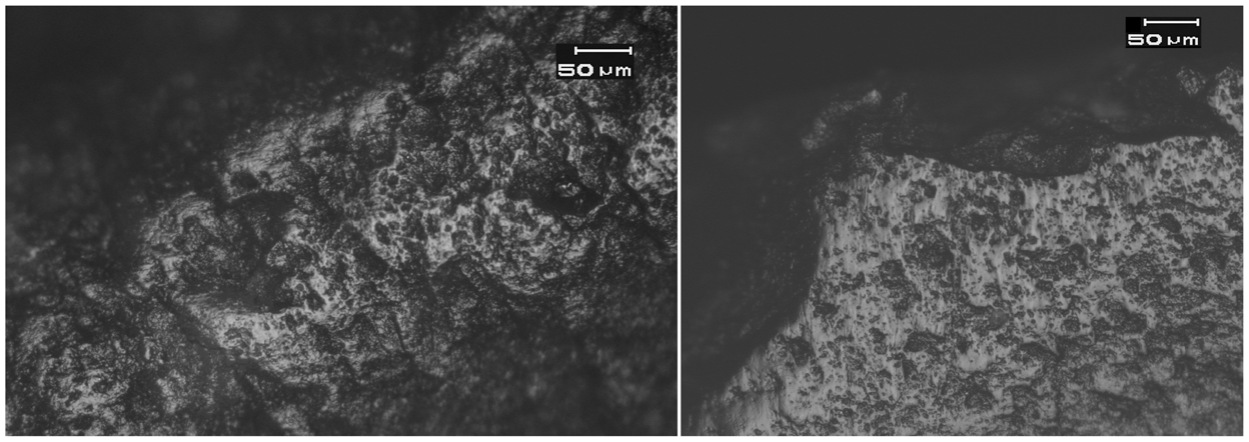
Left: Bright domed wood-working traces on experimental flake tool used as a chisel and scraper. **Right**: Bright, domed wood-working microwear traces on the distal end of EPPNB *tranchet* chisel N19c from the Motza site (SN 14, Locus 4014, Basket 40210). See Figure 2C for location. Magnification is 187.5x, and scale is 50 microns for both images.

**Figure 5:**
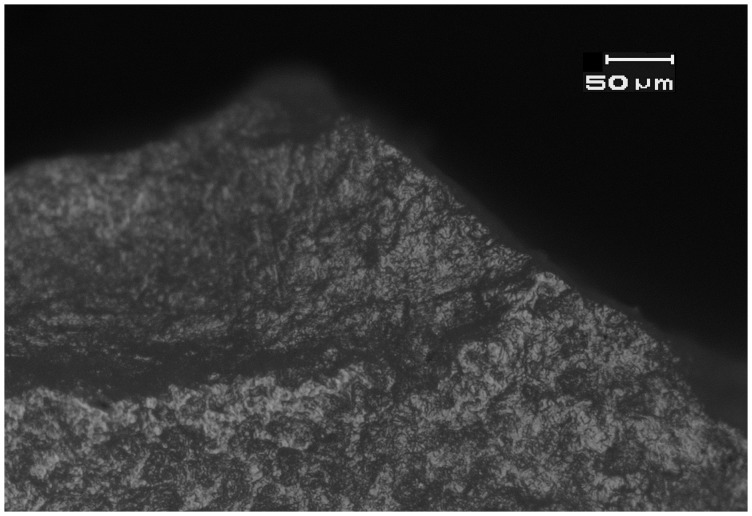
Bright, domed wood-working microwear traces on the distal end of a *tranchet* spall from the Motza site (SN 26, Basket 40309). See Figure 2D for location.

**Figure 6:**
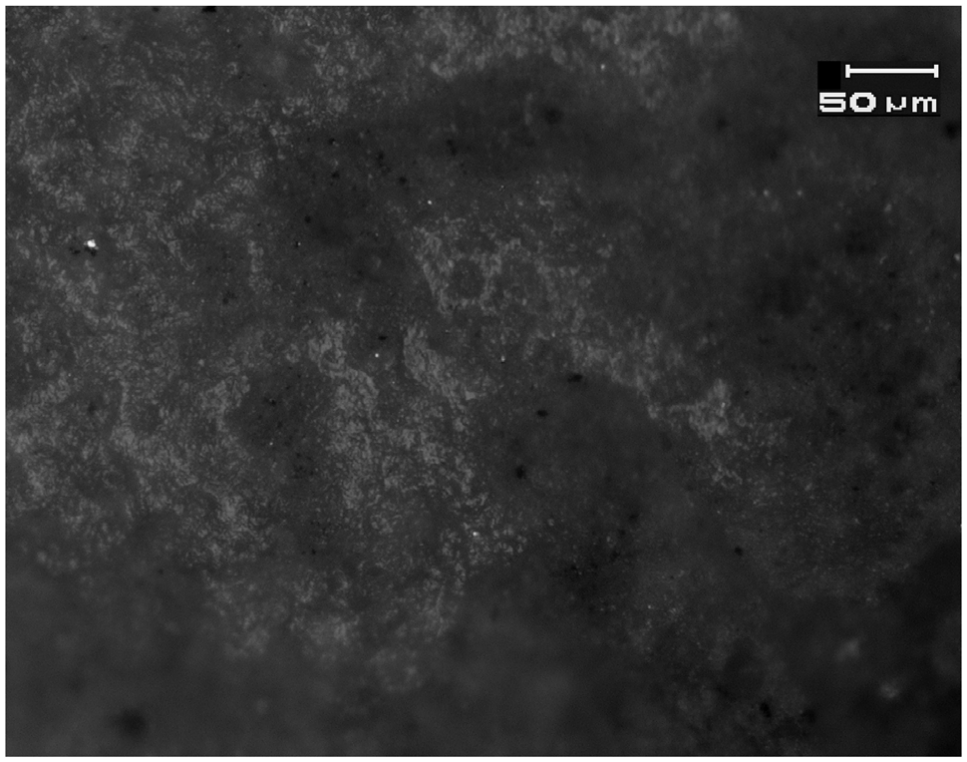
Bright, domed wood-working microwear traces on the distal end of a *tranchet* flint axe with a polished bit from Motza (SN 15, Locus 5016, Basket 50135). See Figure 2E for location. Magnification is 187.5x, and scale is 50 microns for both images.

**Figure 7:**
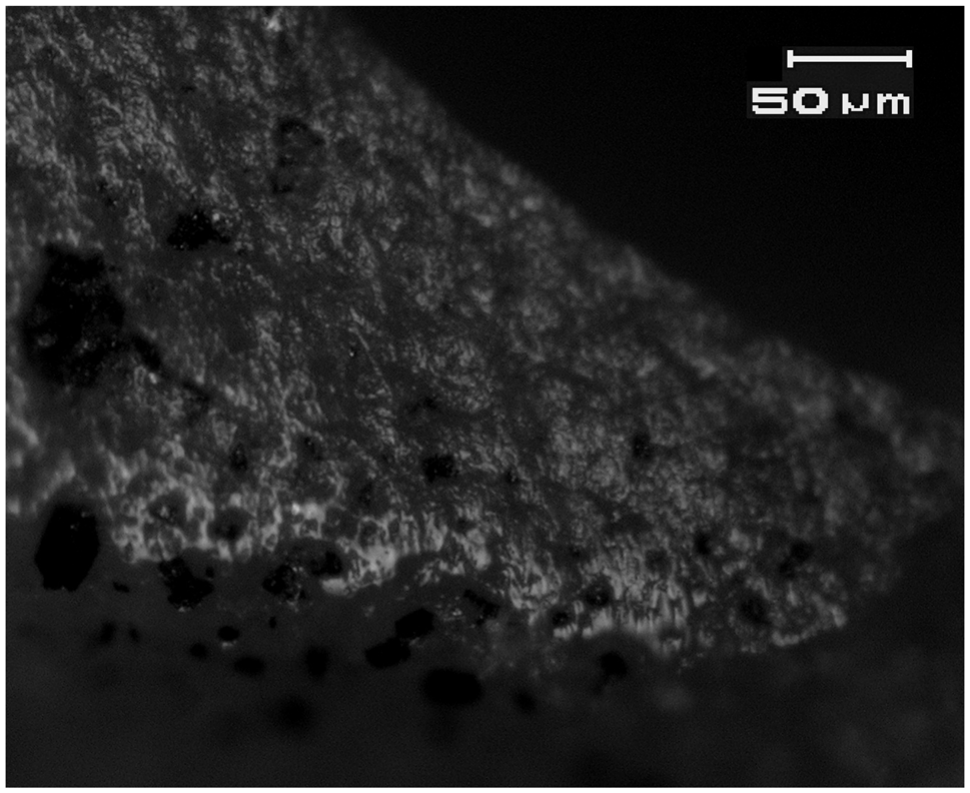
Bright, domed wood-working microwear traces on the distal bit of a flint *tranchet* axe from Motza (SN 34, Locus 5040, Basket 50437). See Figure 2F for location. Axe bit is at the bottom of the photomicrograph.

**Figure 8:**
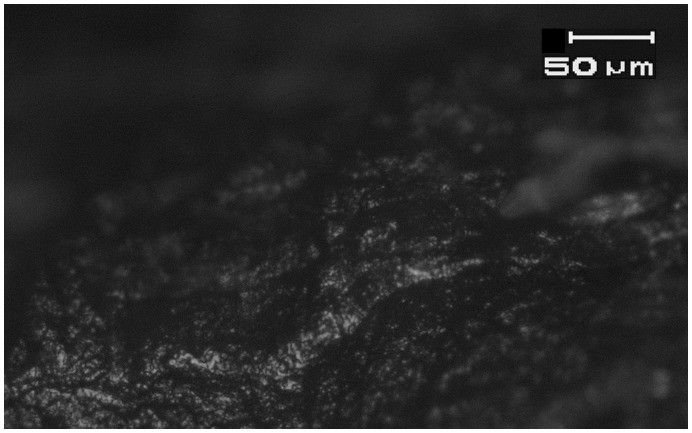
Small patches of bright, domed wood working microwear traces on the distal bit of a flint axeK17c from Motza (SN 11, Locus 5040, Basket 50479). See Figure 2G for location. Magnification is 187.5x, and scale is 50 microns for both images.

Fourteen of the 16 flaked flint axes and chisels in the Motza sample had microwear traces on their edges (88%), and 13 of these (93%) were wood working traces (Table 1, Figs. 3, 4, 5, 6, 7, 8). The only utilized flint biface (SN 33) that was *not* used for wood–working was classified as a recycled biface, probably a flint axe base that was re-used as a stone polishing tool. The lateral edges were worn down and it had large patches of the same stone-on-stone polish that is found on ground and polished groundstone and flint implements (Fig. 1H).

Twelve (75%) of 16 bifacial tools in the Motza microwear sample were complete. Six of the seven complete axes and three of the five complete chisels had hafting traces (Table 1). Two chisel fragments (SN 8, SN 14) had snapped the same way that an experimental chisel broke when hafted in a bone sleeve and used to plane hard wood.

The hafted bifacial tools could have been used elsewhere, discarded at the Motza site, and replaced in their hafts with newly made axes and chisels [28]. However, almost all of the bifacial tools, *tranchet* spalls, and bifacial reduction flakes were made from nodules of light gray or brown fine-grained local Judean flint found in Cenomanian/Quaternary formations located near the site [24]. A few of the bifaces and *tranchet* spalls may have been made of other types of flint, but the fact that so many tranchet spalls were made of the same local flint as the bifacial tools suggests that they were produced at the Motza site and were repeatedly resharpened.

Microwear on the seven *tranchet* axes – including the tool with the polished *tranchet* edge (SN 15, see Table 1) revealed they were used to chop, split, and plane wood (Fig. 2B, E, F, Figs. 3 Right, 6, 7). One small *tranchet* axe (SN 32) had bright, domed wood working microwear traces on its bit (Fig. 3 Right) and hafting traces (Fig. 2B).

An oval *tranchet* axe (SN 13) had a “humped” appearance caused by hinge or step fractures that were produced when biface thinning flakes did not extend all the way to the mid-line. It also had some nicks or tiny flake scars on its distal edge, and weakly-developed wood-working microwear (Table 1). The wear traces on another *tranchet* flint axe (SN 34) were more extensive. Bright, domed, wood polish and striations were visible along most of the battered distal edge of this axe, and there were hafting traces along both of its proximal lateral edges (Figs. 2F, 7). One tranchet axe (SN 11) had a wider cutting edge, with small patches of bright, domed wood-working microwear traces (Figs. 2G, 8). Hafting traces were also visible along the proximal left lateral edge on the dorsal face. The edge of this axe seems to have been resharpened.

Five of the seven *tranchet* chisels (71%) were also used for wood working (Table 1), and one chisel (SN 14) had very well-developed and extensive wood polish along its cutting edge (Figs. 2C, 4 Right). The other four utilized *tranchet* chisels had less damage on their edges, and seem to have been used to smooth or plane wood (Table 1). One of the utilized *tranchet* chisels (SN 17) was “double-edged,” it had *tranchet* spalls removed from its proximal and distal ends. Both edges had visible wood-working microwear traces, but very little edge damage (Table 1). Hafting traces were visible on its dorsal face ca. 20 mm back from its distal edge. This chisel seems to have been hafted and used to plane wood. At some point it was removed, reversed in its haft, and then reused. There were no visible usewear traces on another *tranchet* chisel (SN 10) or on the *tranchet* chisel fragment (SN 12) in the EPPNB microwear sample (Table 1).

Twenty *tranchet* spalls struck from *tranchet* axes or chisels made and used at the Motza site were also examined for microwear traces. Six of them (30%) had identifiable microwear traces on their edges (Fig. 2D, Fig. 5; Table 1). A similar proportion (27%) of 41 *tranchet* spalls in the PPNA Netiv Hagdud microwear sample had usewear traces on their edges [23]. All of the Motza spalls with usewear traces were removed from resharpened *tranchet* axes or chisels that had been used to chop or plane wood. The remaining 14 spalls in the microwear sample were probably removed when *tranchet* axes and chisels were being manufactured. In prior microwear studies, it was found that wood-working traces were usually clearer and more developed on the *tranchet* spalls than on the *tranchet* axes in the samples [23]. However, the wear on three of the six *tranchet* utilized spalls from Motza was not very extensive or well-developed (Table 1). The other three had moderately well-developed wear traces.

Only one flint chisel (SN 9) was not shaped by the *tranchet* technique (Table 1). Some cortex was still visible on the dorsal face of this chisel, and while it only had weakly-developed microwear traces on its distal cutting edge, there were hafting traces on its base. As noted earlier, one of the flint *tranchet* axes (SN 15, Fig. 2E, Fig. 6) had its bit resharpened by polishing – the standard procedure for sharpening the edges of flint axes during the MPPNB, LPPNB, PPNC, PN, and Chalcolithic periods in the Levant. This axe (SN 15) was used to chop wood (Table 1). Hafting traces were visible along both proximal lateral edges. The distal edge was battered, but wood-working microwear traces were not well-developed. When the *tranchet* cutting edge was polished and resharpened, some of the wear traces from earlier activities may have been obliterated.

## Discussion

Stone axes are one of the artifact types that define Neolithic cultures. Indeed, the axe, the plow, fire, and grazing animals are considered the essential tools that allowed farmers to fell trees, clear the forests, and tame the wilderness. However, in the southern Levant stone axes were made and used during the PPNA, before true agricultural systems were established [1], [2], [3], [21], [22], [23]. The flint axes and chisels from PPNA and EPPNB sites are light and small, and they seem to have been used for clearing brush, chopping and splitting small logs and tree branches, and carpentry, rather than for felling and splitting large trees and logs. Large impact fractures and point-initiation flake scars found on larger experimental wood-chopping axes and adzes [26] are not present on these smaller axes. The transition to more permanent settlements during the PPNA and EPPNB periods was marked by the appearance of structures that were built with wood beams and poles as well as stone and mud brick. The PPNA–EPPNB wood-working toolkit seems to have been designed primarily for the carpentry needed to construct these structures, but it was modified when the transition to agriculture led to more tree-felling and forest clearance for fields and grazing lands and when more wood was needed for even larger buildings during the later MPPNB, LPPNB and PPNC periods. More wood was also needed to burn limestone to make plaster for house floors and statues. More fire-wood was also required to meet the increased fuel demands of larger settlements, and may have been needed to construct wooden pens for domesticated animals. The new agricultural life ways that emerged at that time (after ca. 8000 cal. BC) were based on more intensive exploitation of the landscape and on changes in the relationships between humans and their environment.

However, William Balée and others [29], [30], [31] have shown that forest clearance does not always lead to degraded landscapes and the loss of wild food and fiber. Sometimes by opening up the forest, habitats for wild plants and animals are improved. Studies by historical ecologists have shown that when traditional foragers and farmers use axes and fire to open up closed canopy forests, the new niches they create provide more sustainable resources than the “pristine” environments that they have modified [8], [29]. Apparently forests were not cleared by Early Natufian hunter-gatherers who had no axes, but this kind of landscape modification and niche construction began during the PPNA and EPPNB periods, when a few domesticated plants and animals may have been added to the broad-spectrum subsistence system of the complex hunter-gatherers of the southern Levant (although direct archaeological evidence for this is still lacking). It was only when true agropastoral economies developed during the later PPNB periods, and forest tracts needed to be cleared for fields and grazing lands, that larger heavy-duty axes with ground and polished bits (and extensive edge damage) were made and used for tree-felling and other more demanding tasks [21], [22]. Larger axes appear when circular or oval PPNA structures were replaced by rectangular EPPNB structures, including some with massive wooden beams and posts [21], [24]. As noted earlier, this was when animals were domesticated, and lumber was probably needed for pens and fences. Even more wood was needed for cooking and heating, and for lime plaster preparation. The EPPNB occupation layer at Motza contained substantial round and rectangular stone and mud brick structures (some with lime plastered floors), and at least two thick stone walls. There is a rich faunal assemblage and a sizable collection of bone tools from secure, well-dated EPPNB contexts. Several human burials were exposed in that layer, and some small animal and human figurines were recovered [24]. These data suggest that stable, EPPNB forager-farmers were not under stress before they were added domesticates to their subsistence system, and were not overexploiting or degrading forests or grasslands [32]. However, the expansion of agropastoral economies during the following MPPNB, LPPNB, and PPNC periods may have outpaced the rejuvenation of cleared forest lands. During the PPNC, in many houses wooden beams disappeared, or were reduced in diameter, and house floors were made of mud rather than lime plaster [21]. When the Levantine Moist period came to an end around the time when the PN period began, and a cold dry climatic anomaly (6600-6000 cal B.C.) disrupted seasonal precipitation patterns, a cycle of dispersion from nucleated Neolithic villages may have been triggered [1], [4], [7]. It was also around this time, during the Yarmukian phase of the PN period, that adzes replaced axes as the primary wood working tool, and later during the Wadi Rabah phase, chisels increase in abundance [21], [22]. The more versatile adze seems to fit in with the changing needs of Pottery Neolithic, with wood working tasks related to providing fire-wood for pottery and lime kilns, and when large axes may no longer have been needed to exploit the shrinking forests of the southern Levant.

The transition to agriculture in the Levant was a long, complex process, and both climate change and land use practices influenced the outcome. Formal and functional changes in stone tools during the PPNA (9700-8550 cal B.C.) begin with the production of bifacial carpentry tools used to build larger, more permanent houses, but after ca. 8000 cal B.C., as the need for fields, grazing lands, lumber, and fuel increased, heavier, polished axes were made and used to clear the forests. However, PPNB forest management practices do not seem to have led to landscape degradation until the end of the PPNC, when a cold, dry climatic anomaly (6600-6000 cal B.C.) may have accelerated the reduction of the woodlands.

## Materials and Methods

The bifacial tools in the Motza EPPNB sample were examined by Yerkes employing the microwear analysis techniques developed by Sergei A. Semenov [33] and refined by Lawrence H. Keeley [34]. Distinctive micropolishes, striations, and damage scars that form on the edges of chipped stone tools when they are used to perform specific tasks (cutting, scraping, etc.) on certain types of materials (bone, wood, hide, etc.) are examined and interpreted. Microscopic examination is conducted at low power with a stereomicroscope under reflected light at magnifications between 6x and 50x, and at high power under incident light at magnifications ranging between 50x and 1500x. Prior to microscopic examination, the bifaces were drawn, and their greatest length, their thickness, and the width of their cutting edges were measured. Edge-angles of the bits were recorded with a goniometer. Technological details (e.g., retouch, edge damage, presence of cortex, evidence for heat-treatment) were also noted (Table 1). The implements were cleaned in an ultrasonic cleaner with “Top Job” detergent. Ran Barkai and Albert M. Pecora III made some replicas of chipped stone axes, adzes, and chisels that Yerkes used in wood-working experiments. The edges of some of the replica bifaces were shaped with tranchet blows, while others were ground with sand and water, following procedures described in the ethnographic literature. The wear traces that were produced on the edges of the experimental stone wood-working tools were compared with microwear traces on bifaces from the EPPNB layer at the Motza site.

Early and Middle Holocene climate reconstructions are from the summary presented by Weninger et al. [4]. Proxy data used in the reconstructions include Greenland GISP2 ice core data, foraminifera ratios in Mediterranean cores LC21 and MD95-2043, Mediterranean Sea sapropel S1(an organic rich sedimentary deposit), Dead Sea lake levels and salinity, and “flash flood” events recorded in δ18O values in Soreq Cave, Israel speleotherms. Details on these proxy data and interpretations can be found in their summary and in related studies [4], [5], [9], [11], [35], [36], [37], [38], [39].
